# The prevalence of hypertension and its associated risk factors among older adults in Ghana

**DOI:** 10.3389/fcvm.2022.990616

**Published:** 2022-12-20

**Authors:** Baozhen Dai, Stephen Addai-Dansoh, Jonathan Aseye Nutakor, Jeremiah Osei-Kwakye, Ebenezer Larnyo, Stephen Oppong, Priscilla Yeboah Boahemaa, Francisca Arboh

**Affiliations:** ^1^Department of Labor and Social Security, School of Public Health, Southeast University, Nanjing, Jiangsu, China; ^2^Department of Health Policy and Management, School of Management, Jiangsu University, Zhenjiang, Jiangsu, China; ^3^School of Computer Science and Telecommunications Engineering, Jiangsu University, Zhenjiang, Jiangsu, China; ^4^Department of Agriculture Policy and Management, School of Management, Jiangsu University, Zhenjiang, Jiangsu, China; ^5^Department of Accounting and Finance, Kwame Nkrumah University of Science and Technology, Kumasi, Ghana

**Keywords:** hypertension, comorbidity, older adult, healthy lifestyle, Ghana

## Abstract

**Background:**

Hypertension is a worldwide health issue that primarily affects the elderly in our society. However, in comparison to the developed world, the prevalence of hypertension is higher in Sub-Saharan Africa.

**Objective:**

This paper examines the prevalence of hypertension and its associated risk factors among older adults in Ghana.

**Methods:**

Using the World Health Organization’s study on global AGEing and adult health (WHO SAGE) Wave 1 cross-sectional data collected *via* in-person structured interviews; paper and pencil interviews (PAPI) from ten administrative regions of Ghana using stratified multistage cluster design from respondents aged 50+ grouped by decade, this study analyzed a nationally representative sub-sample of 3,997 respondents employing binary logistic regression. Odds ratios (OR) and 95% confidence intervals (95% CI) were used to estimate risk factors associated with hypertension (blood pressure ≥ 130/80 mm/Hg).

**Results:**

There was a 53.72% hypertension prevalence rate among older adults. Hypertension prevalence tends to increase with increasing age. The prevalence of hypertension was associated with residency (*B* = −0.18, OR = 0.84, *p* < 0.017), with urban residents being more at risk of hypertension than rural residents. The prevalence of hypertension increased with overweight (*B* = 0.66, OR = 1.94, *p* < 0.001) and obesity (*B* = 0.82, OR = 2.28, *p* < 0.001). The amount of fruit and vegetable intake was insignificant but had an inverse relationship with hypertension prevalence.

**Conclusion:**

This study has shown that demographic and lifestyle factors significantly affect and explain the hypertension risk among older adults. Medical factors, such as chronic diseases, were largely insignificant and accounted for less hypertension prevalence. Therefore, when interpreting test findings in clinical practice, such as hypertension, it is essential to consider demographic and lifestyle factors. In addition, health policies and primary interventions that seek to improve the standard of living, lifestyle, and wellbeing of older adults need to be critically considered moving forward to lower hypertension prevalence among older adults in Ghana.

## 1 Introduction

A well-known risk factor for cardiovascular diseases and chronic kidney disease is hypertension, also known as elevated blood pressure (1, 2). It was estimated that hypertension will affect 1.56 billion people worldwide by 2025, with a 60% increase in global prevalence, as reported in the 2017 global burden of disease (GBD) (3). According to studies in other nations, hypertension prevalence has increased globally due to population growth, aging, and changes in behavioral risks (2). Despite having a lower incidence of hypertension from 2000 to 2010, high-income countries’ prevalence of hypertension decreased by 2.6 percent during this period, leading to more people’s action to fight the condition. While middle- and low-income countries saw a 7.7% increase in the prevalence of the disease over the same decade, only a small improvement in public awareness, treatment, and control occurred (4). In 2015, more than 1 billion adults throughout the world had hypertension, with the majority living in low and middle-income nations (31.5%) rather than in high-income countries (28.5%) (1, 4).

The main driver of the cardiovascular disease epidemic in Africa is hypertension (5). Several African nations have reported a rise in the risk factors for hypertension, leading to a high disease prevalence (6). Furthermore, a systematic review of the awareness, management, and control of hypertension in Africa revealed a low level of hypertensive status awareness. According to McAlister et al. hypertension awareness levels are much lower in Africa than in Europe and North America (7). The latest data indicates hypertension as Ghana’s fifth most common cause of outpatient morbidity (8), which has been attributed to high population growth, an increase in life expectancy, and lifestyle factors (9). Given the rising incidence of hypertension and the need to minimize its effects, early detection and prevention are vital (10), yet hypertension awareness, treatment, and control are poor in the country (11). Researchers found that obesity, excessive alcohol consumption, physical inactivity, and poor diets were linked to the increased prevalence of hypertension in a review of population-based studies on hypertension in Ghana (12). Most of this low awareness has been attributed to the lack of rigorous educational programs on hypertension (8). However, managing hypertension and its complications is challenging in Sub-Saharan nations where providing resource-intensive care is unachievable. Moreover, the impact on healthcare resources increases due to inadequate diagnosis of hypertension and poor blood pressure control in patients diagnosed (13). In addition, nationalized health insurance is still out of reach in various regions of Africa. As a result, most Africans pay for their medical expenses, which are to some extent supplemented by a few free services provided by the government and donor organizations. These organizations primarily treat infectious diseases, with HIV/AIDS control efforts receiving the most significant share of this funding (14).

According to comparisons with the populations of Western developed countries, Ghana’s population is considered to be a youthful population, which is also true of the people of most Sub-Saharan African countries. However, Ghana’s elderly population has increased dramatically in the last four decades, both in percentage and absolute terms, due to lower fertility and mortality (15). As a result, aged people accounted for around 5.7 percent of the country’s overall population in 2015 (16). Even though this number is relatively modest compared to countries in the northern global economy, Ghana has one of the world’s fastest-growing and oldest populations (17). The Ghana ministry of health reported about 50% of all adults presently have hypertension, up from a prevalence of fewer than 5% a generation ago (18) which has necessitated this study. To the authors’ knowledge, studies on hypertension among older adults in middle and low-income countries are lacking, especially in the case of Ghana, and so is the need for this research. This study aims to bridge that gap by examining the prevalence of hypertension and its associated risk factors among older adults aged 50 and above in Ghana.

## 2 Materials and methods

### 2.1 Samples

The data utilized in this research was derived from the Wave 1 study of the World Health Organization’s project on global AGEing and adult health (WHO SAGE). Six middle and low-income countries (China, Mexico, India, South Africa, and Ghana) participated in the SAGE study (19). This study focused on Ghana, and the sample was stratified into ten administrative regions (Ashanti, Brong Ahafo, Central, Eastern, Greater Accra, Northern, Upper East, Upper West, Volta, and Western) and type of locality (urban/rural), resulting in 20 strata and is nationally representative. Participants involved in the survey were interviewed face-to-face using survey methods that required planning and coordination [paper and pencil interview (PAPI)]. The Rose questionnaire, which was used as an instrument in the survey, relied on validated metrics shown repeatedly to be accurate and reliable. Respondents who could not understand English were given local versions of the questionnaires (20). WHO Geneva organized country survey teams to collect primary data, test samples, and ensure the quality of the data (21). The Ghanaian study had a response rate of 83%. The total population was 5,573, of which 1,576 was excluded due to missing data and persons below 50 years, which resulted in 3,997 as the analytical sample.

### 2.2 Measures

#### 2.2.1 Outcome variable

Hypertension was selected as the outcome variable, considering the aim of the study. Consenting participants in all of the survey’s chosen houses measured their blood pressure three times using non-invasive methods. A calibrated mercury sphygmomanometer was used to take the participants’ blood pressure. Participants were required to sit with their backs supported and their legs resting on the floor for five minutes before taking their blood pressure readings. Thirty minutes before the measurement, they had to abstain from smoking and drinking caffeinated beverages. The participant’s arm, used for the measurement, was placed at an equal level with the participant’s heart. Each measurement was 1–2 min separated from one other. The average of three readings was used to determine the diastolic and systolic blood pressures (DBP and SBP). Patients classified as hypertensive had an elevated mean non-invasive blood pressure reading of 130/80 mmHg or higher.

#### 2.2.2 Covariates

Various socio-demographic data was gathered on the participants, such as their educational level, gender, marital status, age, annual household income, and residence. Participants in this study were categorized into groups of four based on their age: age 50–59 years, age 60–69 years, age 70–79 years, and age 80 years and above. According to their level of education, the current study divided participants into seven categories: those with no formal education, those who had less than primary school completed, those who had completed primary school, those who had completed secondary school, those who had completed high school (or equivalent), college/university completed and postgraduate degree completed. Household income was divided into five quintiles, ranging from the lowest to the highest, based on standardized asset possessions and housing characteristics.

#### 2.2.3 Independent variables

##### 2.2.3.1 Self-rated health

The participants’ general health was assessed using the question, “How would you rank your overall health?” The responses included: 1 = very good, 2 = good, 3 = fair, 4 = poor, 5 = very poor. A binary variable was created: 1 (good) = fair, good, or excellent, and 0 (bad) = very poor or poor.

##### 2.2.3.2 Comorbidities

A total of seven other chronic diseases were identified. Participants were asked, “Have you ever been diagnosed by a healthcare professional with the following chronic conditions?” Angina, arthritis, asthma, stroke, diabetes, depression, and chronic lung disease (1 = yes, 0 = no).

##### 2.2.3.3 Lifestyle

Participants’ weight (kg) and height (m) were used to calculate their body mass index (BMI), which was then grouped into three categories: normal weight, overweight, and obesity. Participants were asked how many servings of vegetables and fruit they ate on a typical day, which was categorized into insufficient (< 5) and sufficient (≥ 5) intake. Participants were also asked if they continuously engaged in physical activity for at least 10 min. Finally, participants were asked if they smoked and consumed alcohol.

### 2.3 Statistical methods

A binary logistic regression was conducted to examine whether socio-demographic (gender, age, residence, education, marital status, household income quintile), self-rated health, comorbidities (seven chronic diseases: arthritis, angina, stroke, asthma, depression, diabetes, and chronic lung disease), and lifestyle (BMI, fruit and vegetable intake, engage in physical activity, smoking, and alcohol consumption) had a significant effect on the odds of being hypertensive. The reference category for hypertension was “No,” indicating hypertension’s absence. Prevalence odds ratios (OR) and 95 percent confidence intervals (CIs) were evaluated. Variance inflation factors (VIFs) were calculated to detect multicollinearity between predictors. All predictors in the regression model had VIFs less than 5, indicating that the assumption of reasonable independence among predictor variables was met (22). This study used *p*-values < 0.05 to indicate significance. Stata/SE version 15.0 (StataCorp, College Station, TX, USA) (23) and Intellectus Statistics (24) were used to perform all analyses.

Ethical approval and informed consent: All study procedures were conducted ethically per the code of ethics of the world medical association (declaration of Helsinki). SAGE obtained ethical approval from the Ethical Review Committee of the WHO (RPC146) and the Ethics and Protocol Review Committee of the University of Ghana Medical School (Accra). Informed consent was obtained from all individual participants included in the study.

## 3 Results

The study’s analysis involved 3,997 older adults from Ghana. The characteristics of the study population are represented in Table 1. The majority (53.16%) of all respondents were older men compared to older women (46.84%). The highest proportion of the population was between 50 and 59 years (39.78%), followed by the subsequent age range. Participants involved in this study had 64 years as their mean age. Compared to those who resided in urban areas (40.51%), more than half of the participants lived in rural areas (59.49%). Regarding their educational background, older adults in Ghana with no formal education had the highest proportion of the population (53.24%). In addition, there was a 53.72% (2,147) hypertension prevalence rate among older adults.

**TABLE 1:** Characteristics of the study sample.

Socio-demographic characteristics	*N* = 3,997 *n* (%)	Hypertension	
		No *n* (%)	Yes *n* (%)
**Gender**			
Male	2,125 (53.16)	1,060 (49.88%)	1,065 (50.12%)
Female	1,872 (46.84)	790 (42.20%)	1,082 (57.80%)
**Age range**			
50–59	1,590 (39.78)	791 (49.75%)	799 (50.25%)
60–69	1,119 (28.00)	486 (43.43%)	633 (56.57%)
70–79	901 (22.54)	394 (43.73%)	507 (56.27%)
80+	387 (9.68)	179 (46.25%)	208 (53.75%)
**Residence**			
Urban	1,619 (40.51%)	670 (41.38%)	949 (58.62%)
Rural	2,378 (59.49%)	1,180 (49.62%)	1,198 (50.38%)
**Education**			
No formal education	2,128 (53.24)	975 (45.82%)	1,153 (54.18%)
Less than primary school	452 (11.31)	200 (44.25%)	252 (55.75%)
Primary school completed	402 (10.06)	191 (47.51%)	211 (52.49%)
Secondary school completed	182 (4.55)	78 (42.86%)	104 (57.14%)
High school (or equivalent) completed	693 (17.34)	334 (48.20%)	359 (51.80%)
College/university completed	131 (3.28)	66 (50.38%)	65 (49.62%)
Postgraduate degree completed	9 (0.23)	6 (66.67%)	3 (33.33%)
**Marital status**			
Never married	48 (1.2)	24 (50.00%)	24 (50.00%)
Currently married	2,249 (56.27)	1,117 (49.67%)	1,132 (50.33%)
Cohabitating	34 (0.85)	22 (64.71%)	12 (35.29%)
Separated/divorced	568 (14.21)	253 (44.54%)	315 (55.46%)
Widowed	1,098 (27.47)	434 (39.53%)	664 (60.47%)
**Household income quintile**			
Lowest	796 (19.91)	421 (52.89%)	375 (47.11%)
Q2	779 (19.49)	363 (46.60%)	416 (53.40%)
Q3	804 (20.12)	347 (43.16%)	457 (56.84%)
Q4	822 (20.57)	363 (44.16%)	459 (55.84%)
Highest	796 (19.91)	356 (44.72%)	440 (55.28%)
**Self-rated health**			
Good	3,365 (84.19)	1,593 (47.34%)	1,772 (52.66%)
Poor	632 (15.81)	257 (40.66%)	375 (59.34%)
**Angina**			
Yes	133 (3.33)	58 (43.61%)	75 (56.39%)
No	3,864 (96.67)	1,792 (46.38%)	2,072 (53.62%)
**Arthritis**			
Yes	530 (13.26)	227 (42.83%)	303 (57.17%)
No	3,467 (86.74)	1,623 (46.81%)	1,844 (53.19%)
**Asthma**			
Yes	147 (3.68)	68 (46.26%)	79 (53.74%)
No	3,850 (96.32)	1,782 (46.29%)	2,068 (53.71%)
**Stroke**			
Yes	84 (2.1)	26 (30.95%)	58 (69.05%)
No	3,913 (97.9)	1,824 (46.61%)	2,089 (53.39%)
**Diabetes**			
Yes	155 (3.88)	54 (34.84%)	101 (65.16%)
No	3,842 (96.12)	1,796 (46.75%)	2,046 (53.25%)
**Depression**			
Yes	65 (1.63)	30 (46.15%)	35 (53.85%)
No	3,932 (98.37)	1,820 (46.29%)	2,112 (53.71%)
**Chronic lung disease**			
Yes	25 (0.63)	12 (48.00%)	13 (52.00%)
No	3,972 (99.37)	1,838 (46.27%)	2,134 (53.73%)
**Body mass index (BMI)**			
Normal weight (< 25 kg/m^2^)	2,857 (71.48)	1,467 (51.35%)	1,390 (48.65%)
Overweight (25–29.9 kg/m^2)^	751 (18.79)	264 (35.15%)	487 (64.85%)
Obese (≥ 30 kg/m^2)^	389 (9.73)	119 (30.59%)	270 (69.41%)
**Fruit and vegetable intake**			
Insufficient	3,858 (96.52)	1,787 (46.32%)	2,071 (53.68%)
Sufficient	139 (3.48)	63 (45.32%)	76 (54.68%)
**Engage in physical activity**			
Yes	72 (1.8)	25 (34.72%)	47 (65.28%)
No	3,925 (98.2)	1,825 (46.50%)	2,100 (53.50%)
**Smoking**			
Yes	1,038 (25.97)	516 (49.71%)	522 (50.29%)
No	2,959 (74.03)	1,334 (45.08%)	1,625 (54.92%)
**Alcohol**			
Yes	2,343 (58.62)	1,119 (47.76%)	1,224 (52.24%)
No	1,654 (41.38)	731 (44.20%)	923 (55.80%)

Older adults in Ghana currently married made up more than half of the study population (56.27%). The household income quintile did not vary much across the lowest to the highest quintile. Furthermore, the majority of the participants (84.19%) reported good self-rated health, 3.33% reported angina, 13.26% reported arthritis, 3.68% reported asthma, 2.1% reported stroke, 3.88% reported diabetes, 1.63% reported depression, and 0.63% reported chronic lung disease. Most participants (71.48%) were of normal weight. About ninety-seven percent of the participant reported insufficient intake of fruit and vegetables. In contrast, just 1.8% of the participants engaged in physical activity daily. The findings also show that 25.97% of the participants were daily smokers, and more than half (58.62%) were reported to be heavy drinkers.

Table 2 evaluated the model based on an alpha of 0.05. The overall model was significant, χ^2^(33) = 198.08, *p* < 0.001, suggesting that socio-demographic (gender, age, residence, education, marital status, household income quintile), self-rated health, comorbidities (seven chronic diseases: arthritis, angina, stroke, asthma, depression, diabetes, and chronic lung disease), and lifestyle (BMI, fruit and vegetable intake, engage in physical activity, smoking, and alcohol consumption) had a significant effect on the odds of being hypertensive. Gender, education, and marital status had no association with being hypertensive. The prevalence of hypertension increases with age. The participants’ residency was associated with hypertension prevalence (*B* = −0.18, OR = 0.84, *p* < 0.017). Older adults who resided in urban areas were at more risk of being hypertensive than those in rural areas. The amount of fruit and vegetable intake was insignificant but had an inverse relationship with hypertension prevalence.

**TABLE 2:** Logistic regression results for association between hypertension and its associated risk factors.

Variable	*B*	SE	χ^2^	*p*	OR	95.00% CI
(Intercept)	0.17	0.44	0.15	0.699	–	–
**Gender**						
Male					1	
Female	0.03	0.09	0.15	0.702	1.03	(0.87, 1.23)
**Age range**						
50–59					1	
60–69	0.25	0.08	9.24	0.002	1.29	(1.09, 1.51)
70–79	0.20	0.09	4.75	0.029	1.23	(1.02, 1.47)
80+	0.10	0.13	0.63	0.429	1.11	(0.86, 1.42)
**Residence**						
Urban					1	
Rural	-0.18	0.08	5.67	0.017	0.84	(0.72, 0.97)
**Education**						
No formal education					1	
Less than primary school	0.06	0.11	0.27	0.603	1.06	(0.85, 1.31)
Primary school completed	-0.09	0.12	0.55	0.460	0.92	(0.73, 1.16)
Secondary school completed	0.03	0.17	0.03	0.871	1.03	(0.73, 1.44)
High school (or equivalent) completed	-0.11	0.10	1.12	0.290	0.90	(0.74, 1.10)
College/university completed	-0.29	0.20	2.17	0.141	0.75	(0.51, 1.10)
Postgraduate degree completed	-1.10	0.72	2.31	0.128	0.33	(0.08, 1.37)
**Marital status**						
Never married					1	
Currently married	0.004	0.30	0.00	0.989	1.00	(0.56, 1.80)
Cohabitating	-0.50	0.47	1.14	0.285	0.61	(0.24, 1.52)
Separated/divorced	0.13	0.31	0.18	0.668	1.14	(0.62, 2.08)
Widowed	0.30	0.30	0.97	0.324	1.35	(0.74, 2.44)
**Household income quintile**						
Lowest					1	
Q2	0.22	0.10	4.43	0.035	1.24	(1.02, 1.53)
Q3	0.33	0.10	9.63	0.002	1.38	(1.13, 1.70)
Q4	0.22	0.11	3.95	0.047	1.24	(1.00, 1.54)
Highest	0.08	0.12	0.38	0.538	1.08	(0.85, 1.37)
**Self-rated health**						
Good					1	
Poor	0.23	0.09	5.87	0.015	1.25	(1.04, 1.50)
**Angina**						
No					1	
Yes	-0.07	0.19	0.13	0.719	0.94	(0.65, 1.35)
**Arthritis**						
No					1	
Yes	0.03	0.10	0.08	0.781	1.03	(0.85, 1.25)
**Asthma**						
No					1	
Yes	-0.09	0.18	0.24	0.627	0.92	(0.65, 1.30)
**Stroke**						
No					1	
Yes	0.50	0.25	4.18	0.041	1.65	(1.02, 2.68)
**Diabetes**						
No					1	
Yes	0.27	0.18	2.24	0.135	1.31	(0.92, 1.87)
**Depression**						
No					1	
Yes	-0.09	0.26	0.11	0.740	0.92	(0.55, 1.53)
**Chronic lung disease**						
No					1	
Yes	-0.07	0.41	0.03	0.866	0.93	(0.41, 2.10)
**Body mass index (BMI)**						
Normal weight (< 25 kg/m^2^)					1	
Overweight (25–29.9 kg/m^2^)	0.66	0.09	54.20	< 0.001	1.94	(1.62, 2.31)
Obese (≥ 30 kg/m^2^)	0.82	0.13	43.45	< 0.001	2.28	(1.78, 2.91)
**Fruit and vegetable intake**						
Sufficient					1	
Insufficient	-0.04	0.18	0.05	0.826	0.96	(0.68, 1.36)
**Engage in physical activity**						
Yes					1	
No	-0.50	0.26	3.73	0.053	0.60	(0.36, 1.01)
**Smoking**						
No					1	
Yes	0.02	0.08	0.08	0.776	1.02	(0.87, 1.20)
**Alcohol**						
No					1	
Yes	-0.02	0.07	0.09	0.764	0.98	(0.85, 1.12)

χ^2^(33) = 198.08, *p* < 0.001. Statistically significant (*p* < 0.05). The model results are reported as beta coefficients with 95% confidence intervals (CIs).

Except for the highest household income quintile, all other quintiles were found to be statistically significant with hypertension prevalence. Self-rated health had a significant association with the prevalence of hypertension. The likelihood of being hypertensive in older adults was high among those who reported poor self-rated health. BMI was significantly associated with hypertension prevalence. The risk of hypertension among older adults increased with overweight (*B* = 0.66, OR = 1.94, *p* < 0.001) and obesity (*B* = 0.82, OR = 2.28, *p* < 0.001) than with a normal weight. Among the seven selected comorbidities in this study, only stroke (*B* = 0.50, OR = 1.65, *p* < 0.041) had a significant association with hypertension prevalence. No association was found between older adults who engaged in physical activity and hypertension prevalence, smoking and hypertension prevalence, and excessive intake of alcohol and hypertension prevalence.

## 4 Discussion

It is crucial to examine the prevalence of hypertension and its related risk factors among the elderly in developing countries, especially Ghana, to enable older adults to live healthy lives. The prevalence of hypertension among the study participants was 53.72%, which is way higher than the prevalence rate of 27% reported by the WHO African Region (25). This high prevalence rate could be due to the use of the new guidelines for defining hypertension (≥ 130/80 mm/Hg) in this study. In addition, factors like changes in diet, lifestyle, and the rising prevalence of obesity may contribute to finding a higher prevalence of hypertension in this study (12). Although the finding is in line with a study conducted in a rural community of Barekese in Ghana, where 438 adults aged ≥ 35 had a prevalence rate of 50.9% (26). However, a systematic review and a meta-analysis on the prevalence of adult hypertension in Ghana conducted by Bosu and Bosu (27) found a 27.0% pooled prevalence of hypertension which is relatively lower than the findings of this study but similar to that of Nigeria (28.9%) (28), Cameroon (30.9%) (29), Sub-Saharan Africa (30.0%) (30), Ethiopia (19.6%) (31), and of 44 developing countries (17.5%) (32). Therefore, the prevalence rate of hypertension reported in this study is higher than in previous studies, indicating the need for concern. Hence, developing primary intervention strategies is vital to curb the prevalence of hypertension among older adults.

The findings of this study indicate that hypertension prevalence tends to increase with increasing age, as reported in previously conducted studies (33–35). This could be due to physiological changes in blood vessels as age increases, increased cellular oxidative stress, chronic low-grade inflammation (36), increased responsiveness to sympathetic nervous system stimuli, altered renin-aldosterone relationship, altered renal and sodium metabolism and decreased baroreceptor sensitivity (37). Furthermore, rural-urban variations in hypertension prevalence are inconsistent on the whole. In the analysis of the current study, older adults who resided in urban areas were more likely to be hypertensive than those in rural areas. However, other studies (35, 38) have come to the same conclusion as this study has. This might be explained by Ghana’s structural environment, as many Ghanaians who reside in urban areas suffer from severe psychological stress due to the numerous inconveniences, deadlines, demands, traffic conditions, and disappointments they encounter daily. Studies have revealed that psychological stress is a significant risk factor for hypertension and other cardiovascular disorders, making this likely (39). Therefore, future research on the effects of psychological factors on the incidence of hypertension in Ghana’s urban areas might be helpful.

This study revealed that older adults below the highest income quintile are more likely to be hypertensive. This finding is supported by a longitudinal cohort study (40), which reported lower odds of hypertension among wealthy African Americans, and a meta-analysis study, which found an overall increased risk of hypertension among individuals with low income. Contrary to these findings, a study of low-income Mexican women aged 18–65 found that those with greater earnings, better asset index, and accommodation had significantly higher systolic blood pressure (41). In Ghana, wealthier older adults are more likely to have personal doctors and adopt regular checkups at the best healthcare centers. This explains why they are at less risk of being hypertensive than older Ghanaian adults below the highest income quintile.

Self-rated health was seen to associate with the prevalence of hypertension in this study. Hypertension was more common in older adults who reported poor health than those who reported good health. The findings of Lewis and Riegel’s (42) study conducted in Southeastern Pennsylvania among 60 years and older adults agree with this result. Many older persons with high blood pressure viewed their health as bad in their study. This could be explained by the physical inactivity of Ghanaian older adults, as revealed in the study results and previously shown to be associated with older adults perceived health (43). Being hypertensive increases the likelihood of having a stroke among older adults in this current study. As previously observed, hypertension is responsible for 51% of global stroke deaths, and local stroke incidence rates correlate with hypertension prevalence (44). This could be explained as high blood pressure adds to the heart’s workload; it damages the arteries and organs over time, causing a stroke (45). According to the clinical research findings, better blood pressure control may help avoid stroke (46).

Chuka et al. in their study, demonstrated a significant association between hypertension among adults residing in Arab Minch with BMI (47). Their finding is consistent with this study which revealed that the prevalence of hypertension increased with overweight and obese compared to those with normal weight. In addition, this finding aligns with previous studies (33, 35, 48, 49). Diet plays an integral part in hypertension prevention. The main components of the dietary factor for hypertension prevention are increased fruit and vegetable consumption (50). However, this study revealed otherwise, with the amount of fruit and vegetable intake having no significant association but an inverse relationship with hypertension prevalence. This could be explained as they are usually consumed in a preserved or cooked form rather than eaten raw among Ghanaians, such as salads (51). Therefore, adding salt during cooking and preservation procedures might counteract the vegetables’ inherent blood pressure-lowering effect. This may have a considerable impact on the prevalence of hypertension and could have concealed the association with fruit and vegetable intake. Although accurate measurement of salt intake is problematic, further studies which examine the association between vegetable intake and hypertension in Ghana or other Western African samples, with appropriate adjustment for salt intake, would be necessary to clarify this question. The inverse relationship between fruit and vegetable consumption and hypertension among participants indicates the beneficial effects of older adults’ consumption of more fruit and vegetables. Therefore, suggesting that, eating adequate amounts of fruits and vegetables impacts the incidence of being hypertensive in older adults.

This study had some limitations and strengths. This research covered all the regions in Ghana, concentrating on face-to-face interviews. As such, the generalizability of these results could be well guaranteed. This study excluded duration of sleep, salt, and red meat intake, which can significantly contribute to the development of hypertension. Again, a cause-effect relationship cannot be attributed because this is not an experimental study.

## 5 Conclusion

This study demonstrated that demographic and lifestyle factors significantly impact and explain the hypertension risk among Ghana’s older adults. Therefore, a better lifestyle should be promoted instead of concentrating on treating illness and its effects, emphasizing disease prevention. The results of this study show that improving older adults’ quality of life and choosing healthier lifestyle habits, like eating a nutritious diet and keeping an average body weight, can lead to improved cardiovascular health. Medical factors, such as chronic diseases, were mainly insignificant and accounted for less hypertension prevalence, while demographic and lifestyle factors were significant and explained the prevalence of hypertension. Therefore, when interpreting test findings in clinical practice, such as hypertension, it is essential to consider demographic and lifestyle factors. Additionally, the government must implement public policies and health initiatives that support an improved standard of living and a healthy way of life among older adults. Future research should include factors such as sleep duration, salt, and red meat intake, which can also contribute to hypertension development.

## Data availability statement

Publicly available datasets were analyzed in this study. This data can be found here: https://www.icpsr.umich.edu/web/NACDA/studies/31381. The Study on Global AGEing and Adult Health (SAGE) Wave 1 was conducted by the World Health Organization.

## Ethics statement

The studies involving human participants were reviewed and approved by the World Medical Association (Declaration of Helsinki). SAGE obtained ethical approval from the Ethical Review Committee of the WHO (RPC146) and from the Ethics and Protocol Review Committee of the University of Ghana Medical School (Accra). The patients/participants provided their written informed consent to participate in this study.

## Author contributions

SA-D, BD, and JN: study conception and design. SA-D and BD: writing original draft. SA-D, JN, JO-K, and EL: acquisition and interpretation of data. JO-K, EL, SO, PB, and FA: provides statistical guidance. BD: supervision and funding acquisition. SA-D, BD, JN, JO-K, EL, SO, PB, and FA: writing—review and editing. All authors read and approved the final manuscript and agree to be accountable for all aspects of the work.
